# State-of-the-Art Fast Healthcare Interoperability Resources (FHIR)–Based Data Model and Structure Implementations: Systematic Scoping Review

**DOI:** 10.2196/58445

**Published:** 2024-09-24

**Authors:** Parinaz Tabari, Gennaro Costagliola, Mattia De Rosa, Martin Boeker

**Affiliations:** 1 Department of Informatics University of Salerno Fisciano Italy; 2 Institute for Artificial Intelligence and Informatics in Medicine, Medical Center rechts der Isar, School of Medicine and Health Technical University of Munich Munich Germany

**Keywords:** data model, Fast Healthcare Interoperability Resources, FHIR, interoperability, modeling, PRISMA

## Abstract

**Background:**

Data models are crucial for clinical research as they enable researchers to fully use the vast amount of clinical data stored in medical systems. Standardized data and well-defined relationships between data points are necessary to guarantee semantic interoperability. Using the Fast Healthcare Interoperability Resources (FHIR) standard for clinical data representation would be a practical methodology to enhance and accelerate interoperability and data availability for research.

**Objective:**

This research aims to provide a comprehensive overview of the state-of-the-art and current landscape in FHIR-based data models and structures. In addition, we intend to identify and discuss the tools, resources, limitations, and other critical aspects mentioned in the selected research papers.

**Methods:**

To ensure the extraction of reliable results, we followed the instructions of the PRISMA-ScR (Preferred Reporting Items for Systematic Reviews and Meta-Analyses extension for Scoping Reviews) checklist. We analyzed the indexed articles in PubMed, Scopus, Web of Science, IEEE Xplore, the ACM Digital Library, and Google Scholar. After identifying, extracting, and assessing the quality and relevance of the articles, we synthesized the extracted data to identify common patterns, themes, and variations in the use of FHIR-based data models and structures across different studies.

**Results:**

On the basis of the reviewed articles, we could identify 2 main themes: dynamic (pipeline-based) and static data models. The articles were also categorized into health care use cases, including chronic diseases, COVID-19 and infectious diseases, cancer research, acute or intensive care, random and general medical notes, and other conditions. Furthermore, we summarized the important or common tools and approaches of the selected papers. These items included FHIR-based tools and frameworks, machine learning approaches, and data storage and security. The most common resource was “Observation” followed by “Condition” and “Patient.” The limitations and challenges of developing data models were categorized based on the issues of data integration, interoperability, standardization, performance, and scalability or generalizability.

**Conclusions:**

FHIR serves as a highly promising interoperability standard for developing real-world health care apps. The implementation of FHIR modeling for electronic health record data facilitates the integration, transmission, and analysis of data while also advancing translational research and phenotyping. Generally, FHIR-based exports of local data repositories improve data interoperability for systems and data warehouses across different settings. However, ongoing efforts to address existing limitations and challenges are essential for the successful implementation and integration of FHIR data models.

## Introduction

### Background

In informatics, operations and data structures can be described by a set of concepts called data models. Because structures and data points need to be connected to represent connections, data modeling offers a visual representation of the system, in a whole or in some parts. For instance, one of the most used conceptual data models is the entity relationship model which is generally linked to a relational database [1]. Data modeling is a process that defines how the data should be maintained in a database. Data types, constraints, relationships, and metadata definitions are among the features specified by a data model [2]. Data models are also crucial for clinical research as they enable researchers to fully use the vast amount of clinical data stored in medical systems. Standardized data and well-defined relationships between data points are necessary to “guarantee reproducible research findings” [3].

Furthermore, data modeling can facilitate interoperability between medical systems. Interoperability refers to the ability to exchange information between computer systems, which is essential in various fields, such as artificial intelligence (AI), big data research and analytics, medical communication, and multinational collaboration. In the medical field, interoperable systems can reduce errors and documentation workload, empower patients, and facilitate information retrieval. In research, real-world information can be collected and used for data mining and AI to generate new hypotheses [4]. The management board of the Healthcare Information and Management Systems Society (HIMSS) defined 3 levels of interoperability: fundamental, structural, and semantic. Fundamental interoperability refers to the communication method between IT firms and devices, while structural interoperability is the format and structure of data being communicated. Semantic interoperability, by contrast, involves the ability of disparate and heterogeneous systems to not only exchange information but also interpret and use it autonomously [5]. Developing a data model would enhance structural and semantic interoperability between medical information systems. Furthermore, efficient data exchange contributes to the reduction of time and financial resources [6].

Health Level 7 (HL7) is a standard-developing organization focused on enhancing information exchange among health care systems. These standards are fundamental in the adoption of electronic health records (EHRs). Fast Healthcare Interoperability Resources (FHIR) is the most recent interoperability standard, preceded by HL7 version 2 and HL7 version 3 [7]. FHIR aims to advance messaging standards to enhance semantic interoperability [8]. Using this standard for clinical data representation is a practical methodology to enhance and accelerate data availability for research. These models can also have the potential to be transformed into other models for analytics purposes [9]. FHIR mapping is the process of identifying the corresponding FHIR resources to real-world data elements. This is an essential step in the FHIR data modeling procedure [10]. When the objective is to maintain semantic interoperability with legacy applications, performing manual data transformations and mappings is necessary to guarantee that the exchanged data are interpreted properly and as expected by all end points [8].

Because not all health care information is structured, there is a need to use other approaches for mapping and FHIR modeling. Natural language processing (NLP) is a branch of AI that deals with the computerized interpretation, representation, and analysis of natural (human) language. In the health care domain, this technology is widely used to interpret and analyze unstructured health data, such as diagnostic reports, medical notes, and prescriptions [11]. The extracted information can then be represented in a structured format, such as a FHIR-based model. In general, it is possible to formalize and integrate unstructured and structured EHR data through a FHIR-based framework [12].

FHIR-based data normalization pipelines are valuable tools in data capture and EHR phenotyping [13]. For instance, a pipeline called NLP2FHIR standardizes unstructured EHR data [14]. Concerning the big data domain, workflows of data harmonization pipelines integrated with FHIR would present a scalable data modeling of large data sets [15]. It is also feasible to use FHIR data models to standardize heterogeneous annotation corpora [16]. All the mentioned potentials will lead to better semantic interoperability between medical systems. To the best of our knowledge, no research has been done so far to comprehensively assess the practical implementations of FHIR-based models and infrastructures. Thus, in this research, we aim to review recent advancements in this field, focusing on the functional data model or structure implementations using this standard. More specifically, this scoping review focused on addressing the question, “What insights can be gained from analyzing the state-of-the-art FHIR-based data modeling approaches considering technological advancements, application in the medical domain, and potential limitations?”

### Objectives

The research objectives are as follows: (1) to provide a comprehensive overview of FHIR-based data models in the context of interoperability, structure, and functionality and summarize the state of the art for developing FHIR-based data models and (2) to highlight limitations, challenges, advantages, and opportunities brought about by FHIR-based data models

## Methods

### Overview

This review was conducted according to the instructions of the PRISMA-ScR (Preferred Reporting Items for Systematic Reviews and Meta-Analyses extension for Scoping Reviews) checklist [17]. This checklist aims to facilitate the development of a deeper comprehension of pertinent terminology, fundamental concepts, and essential items to report for scoping reviews [17]. The checklist is available in Multimedia Appendix 1.

### Study Protocol

We used the PRISMA-P (Preferred Reporting Items for Systematic Review and Meta-Analysis Protocols) 2015 checklist to formulate and draft the review protocol. Protocols for systematic reviews facilitate the organization and recording of review procedures, ensuring the reproducibility of research. In addition, they serve as a safeguard against indecisive judgment during the review process and allow the readers to determine whether selective reporting has been applied [18]. The full checklist and the review protocol are available in Multimedia Appendix 2.

### Eligibility Criteria

To select the papers, we considered the articles that encompass the FHIR standard in the data model development or infrastructure design. The inclusion and exclusion criteria were defined in more detail in Textbox 1.

**Inclusion and exclusion criteria.**

**Inclusion criteria**

- Original articles and case studies from journals and conferences
- Articles related to the Fast Healthcare Interoperability Resources (FHIR)-based data models and structures focusing on a health care condition or using real-world patient data, registries, or data sets
- Articles with high-quality and detailed workflow processes with at least one architecture or data model diagram
- Articles that discuss the barriers, challenges, or limitations of developing FHIR-based data models and infrastructures in a health care domain

**Exclusion criteria**

- Not written in English
- Not accessible
- Letter to the editors, reviews, editorials, commentary articles, short papers without detailed implementation information, posters, and preprint articles
- Not relevant to research questions and objectives; in other words, articles not focusing on FHIR-based data model development or not providing practical and detailed insights into the development or use of FHIR-based data models by a schematic approach
- Papers lacking specific use cases or real-world data sources (practical implementations) or without discussion of limitations and challenges

### Information Sources and Search Strategy

We searched academic databases, such as PubMed, Scopus, Web of Science (standard selection of databases—Web of Science Core Collection), IEEE Xplore, and the ACM Digital Library in May 2023.

The search was conducted using database-specific variants of the basic search term ([“fhir”] AND [“data model” OR “modelling” OR “minimum data set” OR “data element”]) with their synonyms, variations, and full forms.

It is worth mentioning that no time limit was applied to the search to obtain a comprehensive overview of all published articles in this field. We should clarify that the initial pages of Google Scholar (9-10 pages) were investigated as a supplement to the mentioned academic libraries to retrieve additional papers. Full searches are available in Multimedia Appendix 3.

### Study Selection

In a stepwise process, 2 coauthors (PT and MDR) independently screened the retrieved articles and selected the initial studies by applying the inclusion and exclusion criteria to the titles or abstracts or, in some cases, full texts (by rapid skimming). Inconsistencies in the selection were discussed with other coauthors until a consensus was reached. EndNote (Endnote X9; Bld 12062) software was used for article screening and investigation in each step. The full texts of the initially selected articles were assessed in the next phase to check compliance with the eligibility criteria. PT thoroughly reviewed the articles and then discussed with other authors about inclusions. Disagreements were resolved after group discussions.

Each selected study was thoroughly investigated for the appropriateness and clarity of the research methodology and design. We also assessed them to ensure alignment with the study objectives. The rigor of the methods, tools, and techniques used for FHIR-based architectural design was considered in this phase. The presentation of results and the coherence of model interpretation were also closely examined.

### Data Charting Process and Data Items

Two coauthors (PT and MDR) extracted and analyzed the selected articles and charted the data. The final analysis was thoroughly reviewed and confirmed by other coauthors to ensure reliability and rigor. The collaborative review process among coauthors further enhanced the robustness of the results’ interpretation, ensuring a comprehensive and well-rounded analysis of the gathered evidence. The following information was extracted and collected in a spreadsheet: (1) bibliographic information, such as title, authors, and year of publication; (2) data sources; (3) FHIR resources; (4) data transformation and mapping; (5) standards, tools, terminologies, and models; (6) data validation and evaluation; (7) use case.

### Synthesis of the Results

After extraction, we assessed the information to find themes or categories. Subsequently, we performed a general analysis of the papers, based on the overall technical themes and the medical domains. In addition, any important technologies used most in the included articles were comprehensively presented and discussed afterward. Resource frequency analysis was performed via the investigation and counting of FHIR resources used in each data model and infrastructure to find out which resources were more common in system developments. One of the most important aims of our research was to extract and categorize the implementation limitations mentioned by the researchers. Therefore, these aspects were also addressed subsequently to provide a thorough viewpoint of challenges that future scientists may face.

## Results

### Selection of Sources of Evidence

**Figure 1:**
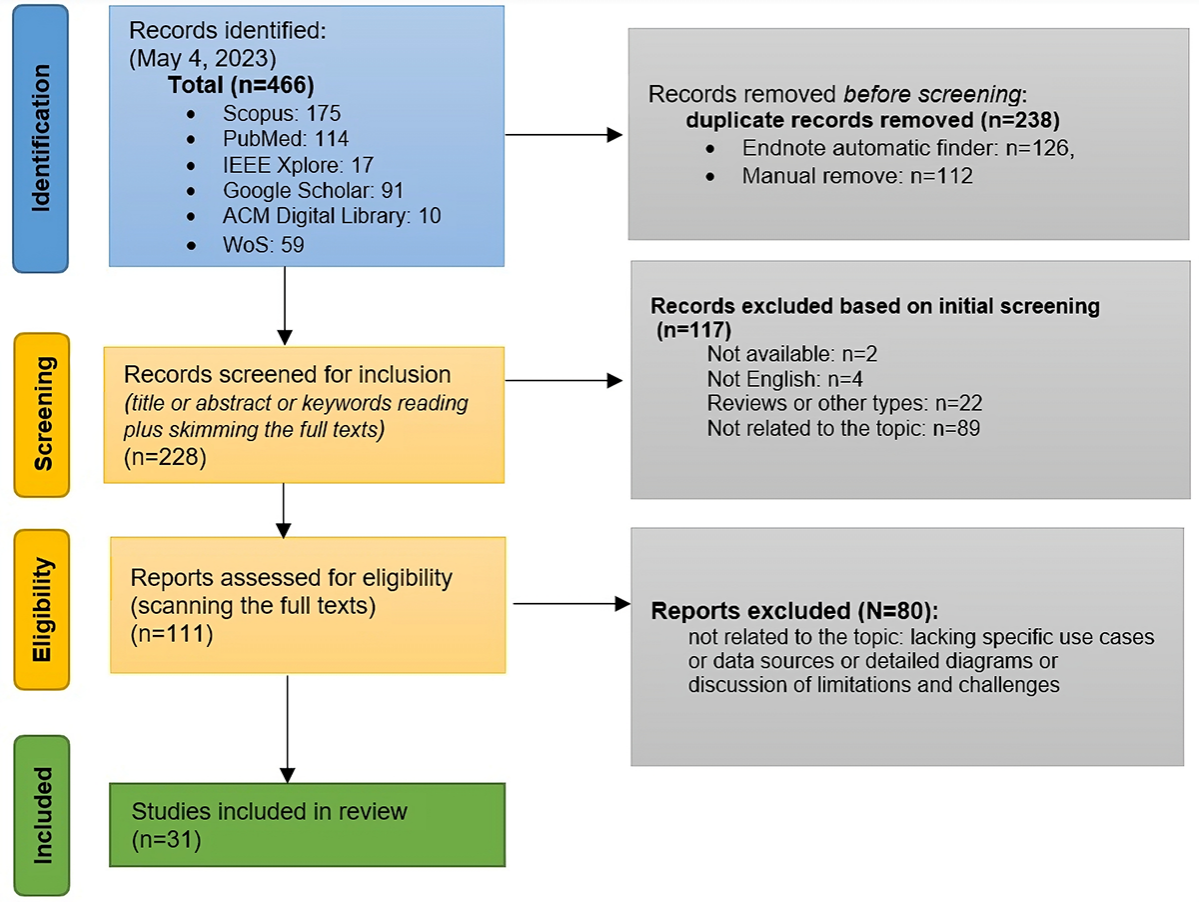
PRISMA (Preferred Reporting Items for Systematic Reviews and Meta-Analyses) flow diagram for study selection. WoS: Web of Science.

Of the overall 466 articles found during the comprehensive search, 238 (51.1%) studies were duplicates. Of the remaining 228 articles, 117 (51.3%) were excluded based on reading titles or abstracts or skimming some full texts. Of the remaining 111 articles for the next phase (full-text assessment), 31 (27.9%) articles were eventually selected to be included in this review. Figure 1 illustrates the PRISMA (Preferred Reporting Items for Systematic Reviews and Meta-Analyses) chart of this study.

### Structural Categorization

After analyzing the full texts of the 31 articles, we categorized them based on 2 models: dynamic (pipeline-based) and static data models.

#### Dynamic Data Models

Data pipelines are chains of functions and activities that lead the input to the output in an attempt for the flow of data to be smooth and automated from source to destination [19]. Dynamic or pipeline-based data models deal with moving, transforming, and analyzing the data using the FHIR standard in their approach. In this category, the FHIR standard has been used as a canonical data model to develop dynamic models. This group encompasses the articles with processes that go beyond static representation and include the movement and transformation of data. Of the 31 articles included, 25 (81%) were related to the development of dynamic data models using the FHIR standard. Table 1 summarizes the extracted information about this category.

**Table 1:** Dynamic models.

Study	FHIR^a^ resources	Data source	Data transformation and mapping	Standards, tools, terminologies, and models	Validation and evaluation	Use case
Lenert et al [9]	Patient, Encounter, Condition, Procedure, MedicationRequest, MedicationAdministration, and Observation	Epic EHR^b^, large academic institution	Flat file to FHIR, FHIR to OMOP^c^, FHIR to PCORnet^d^	HL7^e^ version 2.X, OMOP, PCORnet, Flat file, FHIR CDR^f^, and CDM^g^	By published quality assessment tools	Health care research, especially in the context of COVID-19
Wen et al [20]	Composition and ValueSet	i2b2^h^ Obesity Challenge data set, MIMIC^i^ III obesity discharge summaries	FHIR-based NLP^j^ extensions to CQL^k^, FHIR extensions to NLP2FHIR pipeline	NLP2FHIR pipeline, CQL, NLP engines (cTAKES^l^, MEDXN^m^, MedTime) and PheKB^n^	Phenotype algorithms in PheKB and obesity phenotyping algorithm plus 2 obesity data sets	Obesity
Hong et al [13]	Composition, Condition, MedicationStatement, Procedure and FamilyMemberHistory	i2b2 obesity challenge (discharge summaries)	EHR data to FHIR resources	NLP tools (cTAKES, MedXN, MedTime) and NLP2FHIR pipeline, machine learning algorithms (logistic regression, support vector machine, decision tree, and random forest)	Using MIMIC-III obesity data set as a second data set, evaluation measures (precision, recall, and *F*_1_-score) for performance evaluation	Obesity
Hong et al [14]	Composition, Condition, Observation, Procedure, MedicationStatement, Medication and FamilyMemberHistory	Mayo Clinic’s unstructured EHR data	Unstructured and structured EHR data to FHIR resources	NLP2FHIR pipeline, UIMA^o^ clinical NLP tools (cTAKES, MedXN, MedTime), LOINC^p^, SNOMED CT^q^, RxNorm^r^, and ATC^s^	Reusing annotation corpora, standardizing annotation corpora, NLP2FHIR performance evaluation by precision, recall, and *F*_1_-score	Random notes from EHR
Zong et al [21]	Questionnaire and QuestionnaireResponse	Mayo Clinic patients with colorectal cancer and ACP^t^	Unstructured reports to structured reports and synoptic report to ACP, and ACP FHIR model to CRF^u^	NLP tools and UDP^v^ data sources	Precision, recall, *F*_1_-score, and accuracy	Colorectal cancer
Hong et al [12]	MedicationStatement	Medication data from Mayo Clinic’s EHR	Unstructured EHR data to FHIR, structured data to FHIR resource, and FHIR resources to annotation schemas	NLP tools (cTAKES, MedXN, MedTime), rule-based approach, SNOMED CT, CAS^w^, RxNorm, UIMA, and protégé	Precision, recall, and *F*_1_-score	Random notes from EHR
Williams et al [15]	Patient, Encounter, Observation, Procedure, MedicationRequest, MedicationAdministration, and Condition	MIMIC-IV database for validation	Raw hospital records to AI^x^-friendly and harmonized representation, and database tables to FHIR standard	ETL^y^ framework and Postgres	Openly available MIMIC-IV database to test FHIR-DHP^z^ and syntactic validation of FHIR mapping	Intensive care
Fischer et al [22]	Patient, Encounter, and Observation	German Pulmonary Hypertension registry	CSV file to FHIR bundle collection, source filed names to standard terminology (SNOMED CT, LOINC, ATC, and *ICD*^aa^*-10*) and source data to OMOP schema	ETL process, XSLT^ab^, XPath, OMOP CDM, SNOMED CT, LOINC, ATC, and *ICD-10*	Feasibility assessment by computation time and source data coverage in the target CDM	Pulmonary Hypertension registry
Pfaff et al [23]	Patient, Encounter, Condition, Procedure, Observation, MedicationRequest, and Practitioner	i2b2	CDM to FHIR	CDM and FHIR PIT^ac^	Comparison of generated data by the pipeline and equivalent clinical data of CDWH^ad^ warehouse	Asthma
Rosenau et al [24]	Condition, Observation, Procedure, MedicationStatement, Immunization, DiagnosticReport, and Specimen	GECCO^ae^	Clinical data to FHIR, structured query to FHIR search, and CQL requests	SNOMED CT, LOINC, *ICD-10*-GM^af^, ATC, CQL, and ETL processes	Create test patients and automated and manual test	COVID-19
Zong et al [25]	Observation, Condition, Medication, FamilyMemberHistory, and Patient	Mayo Clinic clinical data warehouse	Clinical entries to FHIR resources, FHIR to RDF^ag^	RDF, classification, machine learning and deep learning, cTAKES, MedXN, MedTime, NLP2FHIR, bag of features, Node2vec, *ICD-9*, RxNorm, and LOINC	Conventional 10-fold cross-validation, AUROC^ah^, and AUPRC^ai^	Cancer
Bennett et al [26]	CodeSystem, ValueSet, MedicationRequest, MedicationDispense, and MedicationAdministration	MIMIC-IV	MIMIC-IV to FHIR	FSH^aj^, py mimic FHIR package, PostgreSQL, and SNOMED CT	Validation by open-source FHIR server (HAPI^ak^ FHIR) by bundles	Intensive care (ED^al^ data)
El-Sappagh et al [27]	Patient, Practitioner, RelatedPerson, Observation, Condition, AdverseEvent, AllergyIntolerance, Location, FamilyMemberHistory, CarePlan, Goal, NutritionOrder, Medication, MedicationRequest, MedicationStatement, Device, Encounter, EpisodeOfCare, CareTeam, and Procedure	WBAN^am^, patient profiles in EHR, and manual data sent by patients	RDB^an^ to FHIR, FHIR to RDB, EHR data to FHIR, and direct mapping of historical data to FASTO^ao^ ontology	FASTO ontology (using FHIR, SSN^ap^, BFO^aq^, and CPG^ar^), OWL^as^ 2, WBAN, CDSS^at^, Protégé, PHR^au^, ISO IEEE 11073, LOINC, SNOMED CT, UoM^av^, FHRBase database, FHIR RESTful^aw^, OAuth2^ax^, SPARQL^ay^, D2RQ platform, Jena API^az^, and Pallet and HermiT reasoners	Ontology is evaluated (assessment of correctness, consistency, and completeness of ontology knowledge) and manual evaluation by experts	Type 1 diabetes mellitus
Zong et al [28]	DiagnosticReport and Observation	ACP and clinical records of Mayo Clinic’s patients	Structured and unstructured data to FHIR-based data profile and directly-inherited data element mapping	CRF, DMM^ba^ model, ETL process, and topic modeling	Precision, recall, and *F*_1_-score	Cancer clinical trials-colorectal
Hong et al [29]	Patient, Observation, Condition, and Procedure	Ovarian cancer database, laboratory test database, and CDM database	Local code to standard code, laboratory test codes to LOINC codes, and mapping between local identifiers and FHIR resource identifiers	Shiny web framework, Shiny apps library, R packages for FHIR data visualization, HAPI FHIR API, LOINC, ICD, and CPT^bb^	Feasibility and adaptability test using public FHIR servers	Ovarian cancer
Hong et al [16]	Condition, FamilyMemberHistory, Procedure, Observation, MedicationStatement, and Medication	Three annotated corpora from SHARPn project, MedXN project, and active Mayo’s clinical NLP project (Family History NLP Project)	Source annotation schemas and FHIR annotation schema	UMLS^bc^, SNOMED CT, LOINC, RxNorm, NLP tasks, support vector machine, annotation tools (Knowtator and Anafora), Protégé ontology editor, and HAPI FHIR API	Evaluation with annotation corpora, calculated precision, recall, and *F*_1_-score	Annotated clinical notes
Marteau et al [30]	Not defined	SHC^bd^ data repositories and Synthea Patient Generator	Map OMOP CDM concepts to FHIR resources by OMOP-on-FHIR (a novel clinical infrastructure)	ETL processes, OMOP CDM, OMOP-on-FHIR, PostgreSQL, psql^be^, SMART^bf^ on FHIR, and Synthea Patient Generator	Qualitative feedback collection and SUS^bg^	Pediatric musculoskeletal disorders
Ismail et al [31]	Patient, Observation, Condition, and Practitioner	MCHHJ^bh^ and CRMHIS^bi^	Data elements to FHIR resources	MongoDB, FHIR RESTful web services, DAO^bj^, Google’s REST console app	User study and questionnaires, generate requests and view responses	Maternal health
Guinez-Molinos et al [32]	Patient, Specimen, DiagnosticReport, and Observation	UC Christus laboratory	Minimum data set fields to FHIR	HAPI FHIR libraries, BPMN^bk^, Cawemo, clinFHIR graphBuilder, JWT^bl^, and MySQL	Performance evaluation (response time, throughput, process management time, main memory storage, secondary storage), and usability test	PCR^bm^ SARS-CoV-2 tests
Burkhardt et al [33]	Patient, Organization, Communication Consent, Questionnaire, QuestionnaireResponse, and CarePlan	Requirement analysis outputs (undergraduate students were surveyed)	Data elements to FHIR	FHIR RESTful API, FHIR Search API, Google’s Flutter, Keycloak, HAPI FHIR, AWS^bn^, Docker, Postgres DB, JWT, and Apache web server	Not stated	COVID-19 symptom tracking
De et al [34]	Patient, Practitioner, RelatedPerson, Organization, HealthcareService, Appointment, Device, Encounter, DocumentReference, AllergyIntolerance, AdverseEvent, BodyStructure, Specimen, Procedure, FamilyMemberHistory, Observation, Condition, Medication, Immunization, CarePlan, ExplanationOfBenefit, and Account	The web-based patient portal at the Mayo Clinic Rochester	Biomedical text to UMLS and patient secure messages to hidden microconcepts	MetaMap, LDA^bo^, multipurpose Annotation Environment, and FHIR definitions	*F*_1_-score to check the consistency between annotators	Random samples of secure patient messages
Liu et al [35]	Condition, Procedure, MedicationStatement, FamilyMemberHistory, Composition, and Bundle	i2b2 2008 obesity data set and MIMIC III data set	Clinical text to FHIR bundle	Deep learning models (text GCN^bp^, GRU^bq^, and CNN^br^), scikit-learn, TensorFlow, Keras, text classification, NLP2FHIR pipeline, cTAKES, and SNOMED CT codes	Accuracy and macroaveraged precision, *F*_1_-score, and recall	Obesity and random notes from discharge summaries
Zong et al [36]	Observation, Condition	Mayo Clinic’s UDP (a clinical data warehouse)	Mappings of report data to 3 data elements (patient clinic number, name, and date of birth) and mappings between elements of PheWAS^bs^ profile and FHIR	UML^bt^, (*ICD-9* and *ICD-10*) codes, LOINC, phecode, Forge editor, FHIR profiling, cross- validation, chi-square distribution associated allelic *P* value, and KS^bu^ test	Cross-validation and FHIR specifications and IGs^bv^	Cancer
Xiao et al [37]	Patient, Encounter, Location, Condition, MedicationStatement, Observation, Procedure, Practitioner, and ConceptMap	MIMIC-III data set (OMOP CDM-based)	OMOP to RDF mappings and OMOP-FHIR mappings	OWL, Protégé, FHIR ShEx^bw^, FHIR RDF, VKG^bx^ (also known as OBDA^by^), MIMIC-OMOP ETL tool, OMOP CDM, Ontop toolkits, SQL, and SPARQL	Using OMOP CDM-based MIMIC-III data set for system evaluation and comparing patient counts identified over MIMIC database and virtual CKG^bz^	Intensive care
Kukhareva et al [38]	Patient, Encounter, Observation, Procedure, and Related Person	Epic EHR	Local codes to LOINC, local codes to standard codes, and QUICK^ca^ to different FHIR versions and profiles	EHR web services, FHIR services, Authorization services, SMART-on-FHIR, native EHR FHIR APIs, SNOMED, and LOINC	Feasibility check by clinicians	Neonatal bilirubin management

^a^FHIR: Fast Healthcare Interoperability Resources.

^b^EHR: electronic health record.

^c^OMOP: Observational Medical Outcomes Partnership.

^d^PCORnet: Patient-Centered Outcomes Research Network.

^e^HL7: Health Level 7.

^f^CDR: Clinical Data Repositories.

^g^CDM: Common Data Model.

^h^i2b2: informatics for integrating biology and the bedside.

^i^MIMIC: Medical Information Mart for Intensive Care.

^j^NLP: natural language processing.

^k^CQL: Clinical Quality Language.

^l^cTAKES: clinical Text Analysis and Knowledge Extraction System.

^m^MedXN: Medication Extraction and Normalization.

^n^PheKB: Phenotype Knowledge Base.

^o^UIMA: Unstructured Information Management Architecture.

^p^LOINC: Logical Observation Identifiers Names and Codes.

^q^SNOMED CT: Systemized Nomenclature of Medicine–Clinical Terms.

^r^RxNorm: medical prescription normalized.

^s^ATC: Anatomical Therapeutic Chemical.

^t^ACP: Australian Colorectal Cancer Profile.

^u^CRF: case report form.

^v^UPD: Unified Data Platform.

^w^CAS: Common Analysis System.

^x^AI: artificial intelligence.

^y^ETL: Extract, Transform, and Load.

^z^DHP: Data Harmonization Pipeline.

^aa^ICD: International Classification of Diseases.

^ab^XSLT: Extensible Stylesheet Language Transformations.

^ac^PIT: Patient data Integration Tool.

^ad^CDWH: Carolina Data Warehouse for Health.

^ae^GECCO: German Corona Consensus Dataset.

^af^ICD-10-GM: International Classification of Diseases–German Modification.

^ag^RDF: Resource Description Framework.

^ah^AUROC: Area Under the Receiver Operating Characteristic Curve.

^ai^AUPRC: Area Under the Precision-Recall Curve.

^aj^FSH: FHIR Short Hand.

^ak^HAPI: HL7 application programming interface.

^al^ED: emergency department.

^am^WBAN: Wireless Body Area Network.

^an^RDB: Relational Database.

^ao^FASTO: FHIR And Semantic Sensor Network based Type 1 diabetes Ontology.

^ap^SSN: Semantic Sensor Network.

^aq^BFO: Basic Formal Ontology

^ar^CPG: clinical practice guideline.

^as^OWL: Web Ontology Language.

^at^CDSS: Clinical Decision Support System.

^au^PHR: personal health record.

^av^UoM: units of measurement.

^aw^REST: Representational State Transfer.

^ax^OAuth: open authorization.

^ay^SPARQL: SPARQL Protocol and RDF Query Language.

^az^API: application programming interface.

^ba^DMM: Dirichlet multinomial mixture.

^bb^CPT: Current Procedural Terminology.

^bc^UMLS: Unified Medical Language System.

^bd^SHC: Shriner’s Children.

^be^psql: a terminal-based front end to PostgreSQL.

^bf^SMART: Substitutable Medical Apps and Reusable Technology.

^bg^SUS: System Usability Scale.

^bh^MCHHJ: Maternal and Child Health Handbook in Japan.

^bi^CRMHIS: Common Requirements for Maternal Health Information Systems.

^bj^DAO: Data Access Objects.

^bk^BPMN: Business Process Model and Notation.

^bl^JWT: JSON Web Token.

^bm^PCR: polymerase chain reaction.

^bn^AWS: Amazon Web Service.

^bo^LDA: latent Dirichlet allocation.

^bp^GCN: graph convolutional network.

^bq^GRU: Gated Recurrent Unit.

^br^CNN: Convolutional Neural Network.

^bs^PheWAS: Phenome-Wide Association Studies.

^bt^UML: Unified Modeling Language.

^bu^KS: Kolmogorov–Smirnov.

^bv^IG: implementation guide.

^bw^ShEx: Shape Expressions Language.

^bx^VKG: virtual knowledge graph.

^by^OBDA: Ontology-Based Data Access.

^bz^CKG: Clinical Knowledge Graph.

^ca^QUICK: Quality Improvement and Clinical Knowledge.

#### Static Data Models

Static models do not follow a sequential or linear flow of data processing; instead, they capture and integrate data in broader aspects and mainly consider data mappings rather than the flow of data. These models are more likely to focus on capturing relationships between variables. They focus on the representation and organization of data within the FHIR standard without necessarily addressing the dynamic aspects of data flow or processing. Out of 31 included articles, 6 (19%) studies were related to the development of static data models. Table 2 summarizes the important information of the articles that presented these data models.

**Table 2:** Static models.

Study	FHIR^a^ resources	Data source	Data transformation and mapping	Standards, tools, terminologies, and models	Validation and evaluation	Use case
González-Castro et al [10]	Observation, Device, Questionnaire, QuestionnaireResponse, FamilyMemberHistory, AllergyIntolerance, Patient, Procedure, MedicationStatement, Condition, and Encounter	Patient medical records, PGD^b^	Map data elements to FHIR resources	SNOMED^c^, and LOINC^d^	Mapping possibilities check	Cancer survivorship (colon and breast cancers)
Montazeri et al [39]	Patient, Observation, Condition, Medication, ServiceRequest, and Practitioner	CPOE^e^ systems, Shafa Hospital (Kerman, Iran)	Data elements to FHIR	CPOE, DigiSurvey platform	Expert panel	Cardiovascular
Shivers et al [40]	AllergyIntolerance, Appointment, CarePlan, Communication, Condition, Consent, CoverageEncounter, HealthcareService, Medication, MedicationAdministration, MedicationStatement, Observation, Patient, Practitioner, Procedure, and ServiceRequest	DAK^f^ data dictionaries that contain core data elements for recommendations about family planning and sexually transmitted infections	Data mappings to FHIR and semantic terminologies (*ICD*^g^*-10*, SNOMED CT^h^, LOINC, and RxNorm^i^)	*ICD-10*, SNOMED CT, LOINC, RxNorm, IG^j^, UMLS^k^, and IPS^l^	Iterative validation of mappings to identify discrepancies gaps, and errors	Family planning and sexually transmitted infections
Lambarki et al [41]	Patient, Organization, Condition, ClinicalImpression, ServiceRequest, Encounter, Observation, Procedure, and MedicationRequest	DKTK^m^	FHIR data elements to corresponding ADT^n^ and ISO standard (11179-3 fields)	*ICD-10*, *ICD-*O-3^o^, TNM^p^, Forge, Simplifier, FHIR validator, clinFHIR, LOINC, ADT/GEKID schema, and OID^q^	FHIR validator to validate FHIR profiles	Oncology
Lichtner et al [42]	Composition, EvidenceVariable, PlanDefinition, ActivityDefinition, Citation, ArtifactAssessment, Evidence, and Group	Members of the COVID-19 evidence ecosystem project (CEOsys)	Model’s items to FHIR resources, information model to EBMonFHIR^r^ resources	EBMonFHIR, CPG^s^-on-FHIR, FSH^t^, SUSHI^u^, HL7^v^ FHIR IG Publisher tool, FHIR core artifacts, GRADE EtD^w^ framework, PICO^x^ framework, Cochrane PICO ontology, SNOMED CT, LOINC, *ICD-10*, ATC^y^, UCUM^z^, CEOsys, FEvIR^aa^ platform	Implementation of a recent COVID-19 guideline recommendation to evaluate EBMonFHIR-based guideline representation	Evidence-based CPG recommendations, COVID-19 intensive care patients’ guideline (evaluation phase)
Khalifa et al [43]	Patient, Practitioner, PractitionerRole, Organization, RiskAssessment, Task, ServiceRequest, MedicationRequest, CarePlan, DeviceRequest, NutritionOrder, SupplyRequest, Questionnaire	Sample reports from ARUP laboratory portal	Genetic laboratory test reports to KDEs^ab^- KDEs to FHIR specification	FHIR profiling, (FHIR CG IG STU1^ac^)	Not mentioned	Genetic laboratory tests

^a^FHIR: Fast Healthcare Interoperability Resources.

^b^PGD: patient-generated data.

^c^SNOMED: Systemized Nomenclature of Medicine.

^d^LOINC: Logical Observation Identifiers, Names, and Codes.

^e^CPOE: computerized physician order entry.

^f^DAK: Digital Adaptation Kit.

^g^ICD: International Classification of Diseases.

^h^SNOMED CT: Systemized Nomenclature of Medicine–Clinical Terms.

^i^RxNorm: medical prescription normalized.

^j^IG: implementation guide.

^k^UMLS: Unified Medical Language System.

^l^IPS: International Patient Summary.

^m^DKTK: German Cancer Consortium.

^n^ADT: Association of Comprehensive Cancer Centres (German).

^o^ICD-O: International Classification of Diseases for Oncology.

^p^TNM: Tumor, Node, Metastasis.

^q^OID: object identifier.

^r^EBMonFHIR: Evidence-Based Medicine on Fast Healthcare Interoperability Resources.

^s^CPG: clinical practice guideline.

^t^FSH: FHIR Short Hand.

^u^SUSHI: SUSHI Unshortens Short Hand Inputs.

^v^Hl7: Health Level 7.

^w^GRADE EtD: Grading of Recommendations Assessment, Development and Evaluation Evidence to Decision.

^x^PICO: Population, Intervention, Comparison and Outcome.

^y^ATC: Anatomical Therapeutic Chemical.

^z^UCUM: Unified Code for Units of Measure.

^aa^FEvIR: Fast Evidence Interoperability Resources.

^ab^KDE: Key Data Elements.

^ac^FHIR CG IG STU1: FHIR Clinical Genomics Implementation Guide–Release 1.

#### Medical Use Case–Specific Summary of Papers

In this phase, we tried to maintain the medical domain consistency in summarizing the articles, and there may be some overlaps between the categories of each article’s health care domain. In the following sections, the included papers are summarized and ordered by specific medical use cases and health care applications.

#### Chronic Diseases

A standard-driven methodology called Clinical Quality Language (CQL) 4NLP was developed to integrate a collection of NLP extensions represented in the HL7 FHIR standard, into the CQL to enhance EHR-driven phenotyping. Using the FHIR standard, specifically the FHIRPath system, enhanced metadata handling and querying by allowing the integration of NLP-derived metadata (such as hypotheticals and negation) into queries. The use case of this research was obesity comorbidities [20]. Another study in the obesity domain used a normalization pipeline to automatically analyze and understand the information in medical records. This FHIR-based approach could detect different sections of medical records and identify important concepts and states of obesity using discharge summaries. The methodology enhanced precise data extraction and portable EHR phenotyping [13]. A similar approach was followed to conduct a case study with obesity data sets. The objective was to predict this condition and the related comorbidities. The sample of adults was categorized into 2 groups called obesity and nonobesity considering their BMI. The design allowed the sharing of deidentified data because only higher-level concepts from knowledge bases and clinical ontologies were included in the FHIR components [35].

In another study, heterogeneous data from a pulmonary hypertension registry were integrated into the Observational Medical Outcomes Partnership–Common Data Model (OMOP CDM) data standard. Common parameters were first identified and mapped to Logical Observation Identifiers Names and Codes (LOINC) and Systemized Nomenclature of Medicine–Clinical Terms (SNOMED CT) as standard terminologies. Extracted data in the form of FHIR bundles were then transformed to OMOP CDM using the Extensible Stylesheet Language Transformations (XSLT). The researchers claimed that FHIR bundles and XSLT can be efficiently and simply used as components of an Extract, Transform, and Load (ETL) process, which can eventually increase data interoperability and applicability [22]. The goal of another research in this area was to map source variables and the value sets to FHIR data elements. The researchers developed a tool called Clinical Asset Mapping Program for FHIR to read Common Data Models (such as informatics for integrating biology and the bedside and Patient-Centered Outcomes Research Network data models) and map the items to FHIR. Using FHIR as a Common Data Model can enhance collaboration, interoperability, and data sharing among health care centers. The clinical use case in the mentioned study was “Asthma” [23].

OMOP-on-FHIR is a technology to convert data elements in OMOP CDM format to the FHIR standard. The researchers used this framework to implement 2 apps to facilitate cohort administration in the context of pediatric musculoskeletal disease research. Accordingly, FHIR can facilitate data access from OMOP CDM databases, support practical integration into health care systems, and enable the development of interoperable clinical applications [30]. For type 1 diabetes mellitus, research presented an ontology-based Clinical Decision Support System based on FHIR and Semantic Sensor Network-Based Type 1 Diabetes Ontology (FASTO). The researchers integrated the FHIR standard, clinical practice guidelines (CPGs), Basic Formal Ontology, and Semantic Sensor Network and implemented a cloud-based interoperable mobile health system for monitoring and managing patients with this condition. Broader adoption and seamless integration within existing EHRs can be achieved through using FHIR and ontology semantics [27]. A multimethod approach involving the development of a Minimum Data Set for cardiovascular computerized physician order entry was presented in another study. The researchers identified and classified critical data elements by reviewing the content of medical records and then mapped them to the FHIR standard. The FHIR standard was used to maintain interoperability between EHR and computerized physician order entry, which can eventually avoid duplicate data entries and redundancies [39].

#### COVID-19 and Infectious Diseases

In the context of COVID-19, clinical data across sites were federated by maintaining a single master patient identifier and consistent demographic information. In addition, this proposed methodology was used to distribute data across networks and maintain common data elements, such as mortality status and social determinants of health data. In the aforementioned approach, the data were loaded into an FHIR Clinical Data Repository, which finally produced real-time linked repositories, including FHIR, OMOP, and Patient-Centered Outcomes Research Network. The researchers found that using FHIR as the initial canonical data model and FHIR subscription protocols for transformation and synchronization of multiple data models has potential benefits for health care research, including the automated creation of research data marts for COVID-19 research [9]. An interoperable platform based on the FHIR standard was developed for convenient reporting and sharing of the polymerase chain reaction SARS-CoV-2 tests across countries. The aim was to create a Minimum Data Set for the tests, followed by modeling associated processes and end points. Implementation continued with standards and interoperability design, software development, testing, and implementation [32]. Another COVID-19–related tool called StayHome was developed for collecting patient-reported outcomes. This reusable mobile app was designed to collect COVID-19 symptoms and share them with health care organizations. The FHIR standard was used to ensure interoperability [33]. In another study on COVID-19, the automatic generation of research ontologies through a terminology server and FHIR profiles was analyzed. The researchers also investigated the process of translating user inputs into FHIR queries. On the basis of the results, it is possible to automatically generate mapping files and ontologies for FHIR-based data and profiles [24]. FHIR-based and evidence-based CPG recommendations for patients with COVID-19 were outlined in another approach. Iterative consensus-based mapping of model elements and links to FHIR correspondences along with modeling of recommendations were covered in the mentioned framework. According to the CPG-on-FHIR architecture, the generated guideline recommendations were represented using FHIR profiles. Using this FHIR-based architecture facilitates the creation of computerized guidelines and their seamless integration into EHR systems [42]. In the fields of family planning and sexually transmitted infections, the researchers structured data dictionaries to improve the mapping procedures to FHIR and multiple terminologies, such as the International Classification of Diseases 10th Revision. The corresponding FHIR resources and codes were then identified and mapped to each data dictionary term. The goal was to prepare inputs (mappings and data dictionaries) for an implementation guide (IG) generation tool and enhance the creation of machine-interpretable guidelines [40]. To clarify, FHIR IG is a collection of guidelines and rules designed to facilitate the adaptation of profiles to align with specific care contexts and promote the standardization of information exchange [44].

#### Cancer Research

In the context of research in cancer clinical trials, FHIR-based pipelines can be used to automatically populate the case report forms (CRFs). The Electronic Data Capture framework was developed in a study to model colorectal cancer trials as a case study. With this strategy, real-world trials can be supported using EHR data [21]. Classification of cancer types and prediction of cancers from unknown primaries were the aims of another research in this field. In the mentioned study [25], genetic data elements (from the oncology reports of patients with cancer) and the associated phenotyping data (from an EHR) were extracted. Researchers presented a network-based infrastructure that modeled the EHR and genetic data with FHIR and Resource Description Framework (RDF) to enhance cancer prediction. In this respect, the performance of different machine learning and deep learning techniques was compared and analyzed [25]. In a paper related to colorectal cancer, data elements were extracted from the CRFs of cancer clinical trials using a data population application. The information was then mapped to an equivalent element in the FHIR cancer profile [28]. An interactive statistics and analysis platform called Shiny FHIR was implemented for ovarian cancer. The system included related R packages (R Foundation for Statistical Computing), FHIR resources, and Shiny (a web application framework). In the FHIR data modeling phase, the ovarian cancer data elements were mapped to corresponding FHIR resources. On the basis of the findings, Shiny can be used in parallel with FHIR to perform interactive analysis [29]. Another interoperable data model called Cancer Survivorship Interoperable Data Elements (CASIDE) was developed in the context of cancer survivorship. The researchers defined data elements and then mapped them to the corresponding FHIR resources. Patient information was illustrated by a collection of FHIR resources to enhance secondary use and sharing of medical data. The research declared the benefits of using FHIR-based models in conjunction with machine learning techniques. In addition, data entry tools can be seamlessly integrated with FHIR-based EHRs [10]. A harmonized data model was also developed in the context of cancer research based on FHIR. German cancer care providers are generally required to report patient data to cancer registries using a specific schema called ADT/GEKID. Therefore, in the mentioned research, the XML representation was compared to the extended version in the German Cancer Consortium (DKTK), and a codification of the cancer life cycle was created. The DKTK FHIR-based data model was represented, and the FHIR resources were identified. Other oncology FHIR profiling efforts were analyzed for reuse in DKTK. It was proved that multiple health care domains can be efficiently modeled using the FHIR standard and that using embedded mapping annotations, FHIR can be smoothly integrated with other standards [41]. The integration of genetic data from heterogeneous sources, including EHR data and genetic reports, was provided using another FHIR-based data model. The objective was to enable the validation of the Phenome-Wide Association Studies results across different institutions using the FHIR-based data profile. The researchers used the developed model to identify cancer genotype-phenotype associations, followed by validation of the associations according to a literature review [36].

#### Random and General Medical Notes

The modeling capability of a data normalization pipeline (NLP2FHIR) was assessed in a study focusing on core clinical resources and unstructured EHR data. The researchers attempted to integrate the unstructured elements to develop an FHIR-based model that successfully standardized the annotated corpora [14]. Another framework was designed to integrate unstructured and structured data into an interoperable format by implementing an NLP-based pipeline using the FHIR-type system. On the basis of the results, the model facilitates the integration of NLP-driven EHR data into a standard FHIR format, supports diverse NLP tools, and provides strong extension capacities [12]. A framework presented in another research for standardizing heterogeneous annotation corpora included 2 main modules (automatic schema mapping module and expert-based annotation and verification module). The system used annotated clinical notes and proved that using FHIR with this kind of heterogeneous data can enhance data reuse as well as integration in medical NLP research [16]. A data model in the context of secure patient messages was developed based on FHIR concepts (related to base, foundation, clinical, and financial categories). The objective was to define significant information contained in these sources. After annotating the sentences and creating a huge corpus, the researchers extracted hidden topics related to 3 microconcepts (fatigue, patient visit, and prednisone as highly discussed topics) through topic modeling. The presented data model could distinguish critical concepts in messages and can be used to identify other narratives on multiple platforms [34].

#### Acute or Intensive Care

In the field of intensive care, researchers aimed to convert the Medical Information Mart for Intensive Care (MIMIC)-IV database elements to FHIR. This database contains patient data from intensive care departments. To support the use of MIMIC-IV on FHIR, a resource demo and a FHIR IG were also created. The benefits of using the FHIR data model are claimed to be its extensive details, which facilitate mappings and conversions of data elements [26]. A FHIR Data Harmonization Pipeline was developed in another study based on an ETL framework. The harmonization of EHR data was performed in 5 phases, including querying the hospital database, mapping the retrieved data to the FHIR format, validating the mapping, transferring the FHIR resources to the patient model database, and exporting the data to the JSON format. Consequently, raw clinical records were transformed into AI-friendly and harmonized representations of data because the hierarchical structure of FHIR may not be sufficiently accessible and standard for AI frameworks. The data could then provide the fast and generic integration of cohort identification methods, facilitating big data processing [15]. In an application for the management of bilirubin in neonates, custom FHIR interfaces were included. After extensive intrainstitutional use, several strategies were explored to modify the app for cross-institutional transfer. Adapting the app for cross-institutional dissemination included clinician-specific implementation using custom FHIR application programming interfaces (APIs), gathering user feedback, differentiating functionality based on FHIR capabilities, implementing gradual replacement with native FHIR interfaces, and using the HL7 Quality Improvement and Clinical Knowledge (QUICK) logical data model for mapping to different FHIR versions and profiles [38]. The QUICK model encapsulates specific details of FHIR (eg, the differences between elements and extensions), enabling a more focused approach to the attributes and classes. This allows for the logical data model specifications to be identified with greater clarity [45]. Another research focused on knowledge graphs (KGs) and semantic modeling. In the mentioned research, the relational databases of the OMOP were used to develop the FHIR-Ontop-OMOP system. The aim was to generate virtual KGs from the databases. The generated KGs were evaluated for the accuracy of data transformation and compatibility with FHIR RDF using an intensive care data set (including medications, vital signs, observations, survival data, and so on). This semantic system could fully represent an OMOP database as an FHIR-compliant representation using KGs, thus enhancing the interoperability of OMOP CDM and FHIR [37].

#### Other Conditions

A study aimed at implementing a maternal health record system with a data access model based on RESTful web services. In the proposed data model, important data elements were mapped to FHIR resources. Maintaining the related data as FHIR resources enhanced interoperability, efficient data exchange, and evidence-based decision-making [31]. Another article dealt with genetic laboratory tests. The researchers aimed to map the test elements to FHIR format based on an IG. FHIR clinical genomic IG is a beneficial and almost comprehensive tool for sharing genetic test results [43].

### Technical Approaches

#### Overview

Concerning developing data models or infrastructures using the FHIR standard, several tools have been used in the reviewed research articles. This section summarizes the important or common tools and approaches. These items include FHIR-based tools and frameworks, machine learning approaches, and data storage and security.

#### FHIR-Based Tools and Frameworks

##### NLP2FHIR Pipeline

**Figure 2:**
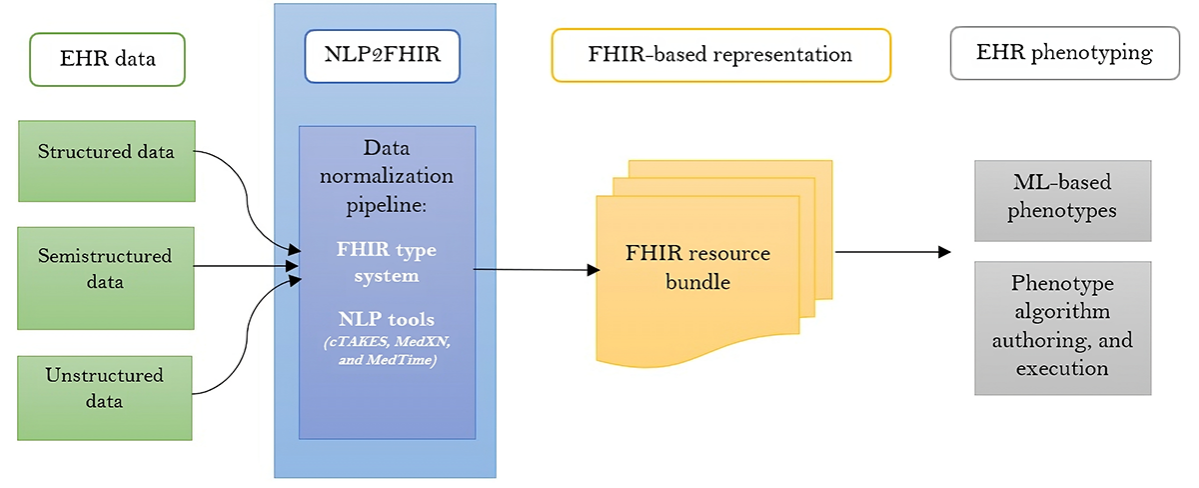
NLP2FHIR data normalization pipeline and its applications [46]. cTAKES: Clinical Text Analysis and Knowledge Extraction System; EHR: Electronic Health Record; FHIR: Fast Healthcare Interoperability Resources; MedXN: Medication Extraction and Normalization; ML: Machine Learning; NLP: Natural Language Processing.

In the field of NLP, there is a FHIR-related clinical data normalization pipeline called NLP2FHIR for EHR data modeling. This pipeline can be used to standardize and integrate structured and unstructured data stored in EHRs. In other words, it can make unstructured EHR data consistent and integrate it with structured data. This procedure facilitates portable EHR-driven phenotyping and large-scale data-driven analytics. Several studies used NLP tools as part of the data model’s implementation. The NLP2FHIR pipeline was used in 5 articles [13,14,20,25,35]. As shown in Figure 2, this pipeline receives the EHR data in various formats (structured, semistructured, and unstructured) as input. The pipeline itself uses the FHIR-type system as well as NLP tools. The binaries required to run this pipeline are MedTagger, clinical Text Analysis and Knowledge Extraction System (cTAKES), MedTime, Medication Extraction and Normalization (MedXN), and Unified Medical Language System Vocabulary and Terminology Service. The raw clinical data are then transformed into FHIR bundles. Phenotypes can be created based on FHIR bundles, and finally, the FHIR-based data are easily integrated into EHR systems [46].

To elaborate more, MedTagger is an open-source NLP pipeline that underpins the implementation and handling of unstructured clinical data. The system differentiates between general NLP processes and task-specific NLP knowledge, enabling experts to directly encode clinical information using relevant terms and phrases [47]. cTAKES is another open-source NLP system that extracts free-text and narrative information from EHRs and enables semantic processing of this information. It is developed based on the OpenNLP toolkit and a framework called Unstructured Information Management Architecture [48]. MedTime is a hybrid framework containing both machine learning and rule-based approaches for the extraction of temporal information from unstructured clinical text. This system has a high performance in recognizing temporal expressions and clinical incidents [49]. MedXN is a system for extracting pharmaceutical information from clinical notes, making it compatible with RxNorm representation with high accuracy [50]. Unified Medical Language System Vocabulary and Terminology Service enables the interaction of the Unified Medical Language System and the different source vocabularies [51].

Using this pipeline, the data contained in discharge summaries can be transformed into FHIR resources [13]. In addition, normalization and mapping rules as well as NLP-based FHIR extensions can be implemented through NLP2FHIR. It is proven that this pipeline can be a practical tool for modeling unstructured data to eventually integrate the structured elements into models [14]. When NLP-derived artifacts are stored as FHIR extension metadata fields through NLP2FHIR, these elements can be seamlessly incorporated into queries. This integration supports more comprehensive and precise querying by including clinically relevant metadata extracted from unstructured data [20]. In a study conducted by Zong et al [25], each entry in family history records was processed by the NLP2FHIR pipeline, which involved identifying and normalizing medical concepts with MedXN, cTAKES, and MedTime tools. Liu et al [35] followed a workflow of tokenizing documents from 2 data sets and improved the embedding performance by preprocessing (eg, removing less frequent words as well as stop words). The JSON-formatted FHIR resources from the NLP2FHIR pipeline were then transferred into token-like representations categorized into FHIR resources and bundles. cTAKES was also used for concept normalization. The researchers compared the performance of models based on the information in this pipeline with models with original texts.

##### Substitutable Medical Apps, Reusable Technology–on-FHIR

This specification can be used for data and security requirements for health-related applications. Substitutable Medical Apps, Reusable Technology (SMART)-on-FHIR defines a workflow of secure requests for data access, as well as receiving and using that data [52]. In other words, this specification is a framework that includes web standards that are used to define health applications based on the FHIR-based data stored in an FHIR server. Marteau et al [30] developed a SMART-on-FHIR application, including a query and an upload page to enhance data organization and accessibility. The research highlights that clinicians and health care professionals can query health care applications through FHIR APIs [30]. The applications containing SMART-on-FHIR can interact and integrate with EHR systems through APIs and provide efficient “plug and play interoperability.” Kukhareva et al [38] discussed the balance between portability and functionality of SMART on the FHIR applications and how the developers should consider this balance. A comprehensive approach with the integration of user-centered and technical methods is needed to optimize this balance.

##### Evidence-Based Medicine–on-FHIR and CPG-on-FHIR

Evidence-Based Medicine–on-FHIR (EBMonFHIR) is a knowledge asset project on FHIR resources for EBM. The objective of EBMonFHIR is to offer interoperability for people who generate, analyze, synthesize, disseminate, and implement clinical evidence and CPGs [53]. CPG-on-FHIR is an IG that uses FHIR resources to build computable and interoperable representations of clinical guideline contents [54]. Lichtner et al [42] developed an IG that used the resources developed by EBMonFHIR to represent primary evidence and the evidence-to-decision process. These resources were eventually integrated into the CPG-on-FHIR framework. Both EBMonFHIR and CPG-on-FHIR are supported by the HL7 Clinical Decision Support staff and represent different aspects of evidence-based guideline recommendations. The former focuses on the justification aspects of the recommendations, while the latter focuses on the implementation aspects of the recommendations.

##### clinFHIR

clinFHIR is a web-based, open-source educational environment that also allows developers to create or search FHIR-based resources [55]. ClinFHIR graphBuilder is used to model the relationships between resources. This tool also assembles resource instances into a graph with related resources to specify a scenario using FHIR [32]. Accordingly, the structure of models can be visualized using clinFHIR software [41].

##### HL7 Application Programming Interface FHIR

HL7 API (HAPI) FHIR is a comprehensive implementation of FHIR in the Java language [56]. The API is available for both FHIR clients and servers [57]. Several studies used HAPI FHIR in the data model implementation process. Bennett et al [26] used the HAPI FHIR server in the process of validation, bulk export, and writing data to NDJSON files. Hong et al [29] used the API to put ovarian cancer data into FHIR resources. They also used the client API to upload structured FHIR data elements to the FHIR server. HAPI was one of the test servers that was used to assess data quality and server stability. The API can also be used in the NLP domain. Hong et al [16] used HAPI FHIR for annotation serialization; they converted the annotations to FHIR XML and JSON formats that were eventually represented in an FHIR-consistent format. The HAPI FHIR resource validator API was also used to validate the resources for compliance with the FHIR specification. In the model presented by Guinez-Molinos et al [32], the HAPI FHIR database was used to store resources, and the HAPI server was responsible for the interoperability layer of the model. The HAPI libraries were also used to construct resources, messages, and end points. For persistent storage of FHIR-based data and as an API server, Burkhardt et al [33] used HAPI FHIR V4.2.0. HAPI generally offers standard functionalities, such as create, read, update, and delete APIs, along with specialized domain-specific tools, including CQL. This capability enables developers to concentrate more on the specific needs of their app.

#### Machine Learning Approaches

Apart from the use of the NLP2FHIR pipeline discussed in the previous section, some other articles used simple NLP tools and algorithms to convert unstructured data into structured data elements adhering to a specific schema for better data description [21]. Hong et al [12] used Unstructured Information Management Architecture NLP tools such as MedXN and MedTime in the normalization phase to enhance interoperability. MedXN was used to extract medication extraction concepts, and MedTime was used to extract FHIR-defined temporal elements. Separate NLP extraction modules were developed to extract information directly from free text for those entities that cannot be extracted by current NLP tools. In a classification system developed by Hong et al [13], 4 machine learning algorithms, including support vector machine (SVM), random forest, logistic regression, and decision tree, were implemented to train the classifiers of the disease prediction module; the features that were used by the system were extracted from FHIR resources as well as terminology extensions. Among all methods, the random forest approach had the best performance. Zong et al [25] analyzed some deep learning and machine learning backbone models to compare the performance of cancer prediction. The bag of features (or bag of words) was used in their research based on the values of attributes in the FHIR model. A graph embedding method called Node2vec was used to learn the patient’s features (a vector). Generally, 3 methods of feature generation were compared, including the bag of features, Node2vec, and the bag of features combined with Node2vec. Moreover, 7 classification algorithms were analyzed and compared (random forest, logistic regression, naive Bayes, deep neural network, SVM, graph convolutional network [GCN], and convolutional neural network). Node2vec+bag of features and random forest classifier showed the best performance. To analyze the potential of integrating the unstructured FHIR data representations into deep learning methods, Liu et al [35] used Gated Recurrent Unit, CNN, Text GCN on NLP2FHIR inputs, and raw text. The results highlighted that the best performance was achieved by using the Text GCN classifier in NLP2FHIR input. Therefore, this combination can enhance interoperable EHR phenotyping.

In the data model presented by Zong et al [28], NLP tools were used to provide structured data for the ETL process from unstructured data (such as surgical reports). To cluster each patient in the patient subgrouping process, a model called Dirichlet Multinomial Mixture was used. In the Dirichlet Multinomial Mixture model, one document represents a single topic, which makes it suitable for clustering short texts. The genetic relationship extractor that was developed by Hong et al [16] used SVM as a learning model; the goal was to extract the “FamilyMemberHistory.relationship” FHIR element. Eventually, the NLP performance of the corpora was analyzed. On the basis of the results, an NLP engine can be developed on a pooled corpora that offers enough annotations to train a model. To learn the concealed topics of patient messages, De et al [34] used latent Dirichlet allocation, an unsupervised topic-learning model. It was claimed that latent Dirichlet allocation is effective in finding common topics with well-known terms but, at the same time, tends to overlook less frequent yet important topics in patient messages.

#### Data Storage and Security

Several studies used PostgreSQL (also known as Postgres) as the database management system [15,26,30,33]. This system is an SQL-based open-source relational database management system that is compatible with JSON document storage. In the study of Williams et al [15], data storage was based on FHIR resource type, and each resource was mapped to a separate JSON structure. Bennett et al [26] used the MIMIC-IV database; the data contained in the data source was loaded into Postgres and the HAPI FHIR server. The data elements were then mapped to JSON within that system. The research indicated a substantial increase in storage requirements when data is converted to FHIR and further when inserted into HAPI FHIR. Specifically, the HAPI FHIR format required significantly more storage space compared to the basic relational structure. To store the “OMOP CDM database” generated by the Synthea synthetic patient generator, Marteau et al [30] used Postgres. The database was then modified to incorporate additional data needed for OMOP-on-FHIR. A PostgreSQL client application (psql) was subsequently used to interact with the database.

Burkhardt et al [33] also used this system along with the Apache web server in their proposed architecture. By contrast, Ismail et al [31] used MongoDB (NoSQL, or nonrelational data storage) for efficient data record manipulation processes. MongoDB can conveniently handle JSON structure, which is the format of the FHIR resources sent and received by servers. Using MongoDB provides a straightforward transformation of JSON objects into JSON documents, making storage and management more efficient. The database can handle FHIR resource searches based on specified criteria. This is facilitated by the MongoDB Data Access Object component that is responsible for validating the JSON strings received from clients.

Some researchers implemented OAuth for security purposes [27] and used Keycloak as an identity provider [33]. JSON Web Token was also used in other studies [32,33] to provide authentication services. This token securely shares information between end points by a JSON object.

### Resource Frequencies

Table 3 illustrates the frequency of each resource in the included articles. It should be mentioned that in this section, we only discuss the official base FHIR resources, and the items mainly considered as profiles, extensions, or domain-specific resources are not illustrated here (eg, “LabTest, Imaging, Referral, Risk, CoverageEligibility, ClaimPayment, ProcedureRequest, Dosage, and DeviceUseStatement” [27,34,38,40]).

**Table 3:** Frequency of Fast Healthcare Interoperability Resources (FHIR) resources mentions in the included articles (n=31).

FHIR resources	Articles, n (%)
Observation	21 (68)
Condition	19 (61)
Patient	18 (58)
Procedure	16 (52)
Encounter	11 (35)
MedicationStatement	10 (32)
FamilyMemberHistory and Practitioner	8 (26)
MedicationRequest and Medication	7 (23)
Composition and CarePlan	5 (16)
MedicationAdministration, Questionnaire, AllergyIntolerance, Organization, and ServiceRequest	4 (13)
QuestionnaireResponse, DiagnosticReport, Specimen, RelatedPerson, Device	3 (10)
ValueSet, Immunization, AdverseEvent, Location, NutritionOrder, Communication, Consent, HealthcareService, and Appointment	2 (6)
CodeSystem, MedicationDispense, Goal, EpisodeOfCare, CareTeam, DocumentReference, BodyStructure, ExplanationOfBenefit, Account, Coverage, ClinicalImpression, EvidenceVariable, PlanDefinition, ActivityDefinition, Citation, ArtifactAssessment, Evidence, Group, Bundle, ConceptMap, PractitionerRole, RiskAssessment, Task, DeviceRequest, and SupplyRequest	1 (3)

### Publication Distribution

**Figure 3:**
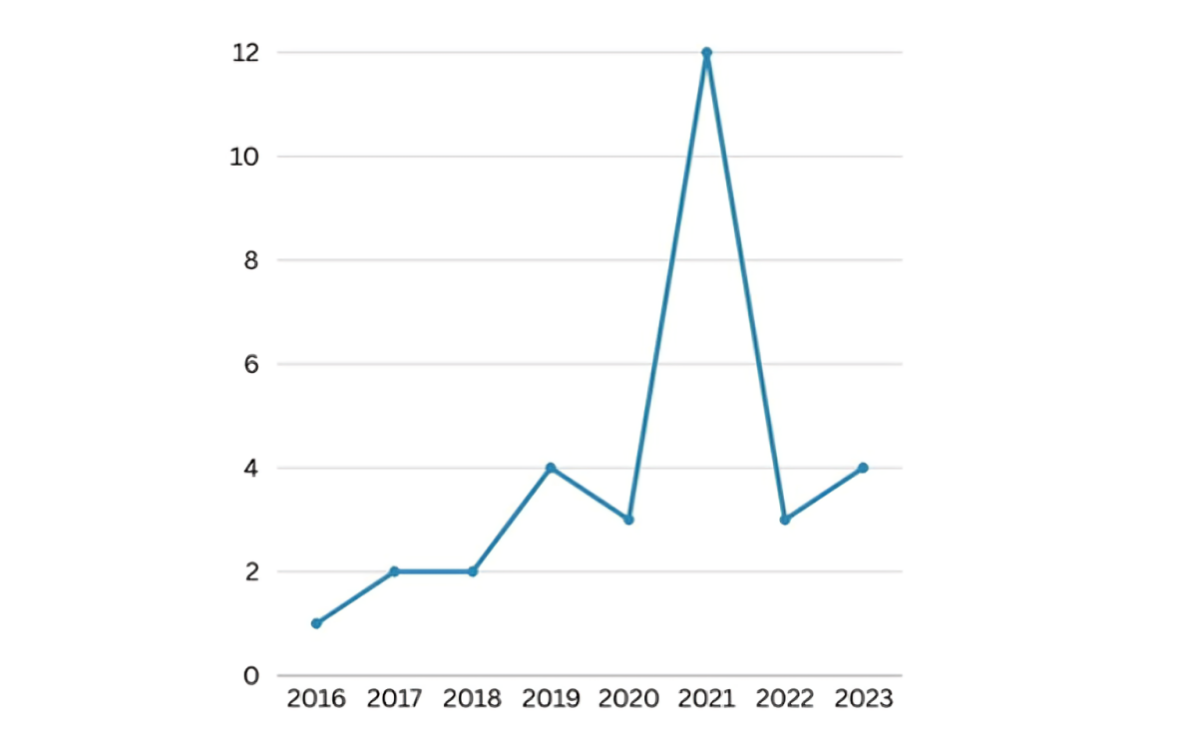
Yearly distribution of reviewed articles.

Figure 3 illustrates the distribution of included studies according to the publication year. As shown in the figure, the year 2021 encompassed the highest number of publications.

### Limitations and Challenges of Developing Data Models

#### Overview

Data model development can present many challenges and constraints arising from issues of data integration, interoperability, standardization, performance, scalability, generalizability, etc. In this section, we discuss the significant challenges and limitations identified in the reviewed papers, which researchers should take into account when developing FHIR-based data models and architectures. It is worth noting that there would be overlaps in the categorization of limitations and challenges.

#### Data Integration

It is the process of combining data from multiple sources and creating a unified data set. The initial stage of working on data analysis, reporting, and forecasting is data integration [69].

Regarding data integration, the problems mentioned in the analyzed papers include requiring some manual ETL processes [9], manual download of FHIR resources [12], reproducibility issues [20], maintaining robustness [9,20], using only 2 data sets and information loss [13], challenging content normalization [14], single CRF with limited data elements and inadequate questions [21], quality and completeness of the database documentation and nonautomatic concept recognition and considerable data preparation process [15], using incomplete synthetic database [30], no data curation during transformation, bias in database [26], storage space costs [26,33], maintaining inadequate aspects of data [27], synchronization issues and hard-coded mapping [22], privacy and confidentiality issues and limited corpora reuse [16], ignoring continuous changing of values over time [25], lack of comprehensive use of health care records due to lack of education and awareness [31], mapping rules based on only 2 use cases and not including information about generic data [10], manual FHIR mapping and reviewing data of only 1 setting [39], the requirement to implement a structured information model to an existing data dictionary [40], issues with ADT data set as a national reference (completeness and accuracy) [41], and no robust mapping [37].

#### Interoperability

Interoperability issues are related to the limitations in the seamless transfer and exchange of information between medical systems or applications [70].

Interoperability issues mentioned in the papers include reproducibility concerns [20], organizational interoperability issues [24], privacy and confidentiality issues [16], synchronization issues and hard-coded mapping [22], no evidence to attain a balance of functionality and portability and dissemination barriers due to development and integration costs [38], and extra mapping effort, which affects flexibility and adaptability of the framework [36].

#### Data Standardization

It is a crucial step in transforming data into a uniform format to enable the shared use of advanced tools and techniques, large-scale analytics, and collaborative research [9,71]. Therefore, standardization issues are related to challenges in attaining standard and consistent data representation across different medical systems.

The issues that could be categorized into the data standardization challenges include difficult rule-changing in compiled java code for data transformation [9]; semantic gaps between NLP system’s data model and FHIR specification [14]; no adequate standardization [12]; mapping accuracy issues [15]; handling valid source system data with no match in FHIR [23]; SNOMED CT postcoordination limitations [24]; not mapping to the US Core as standard ontology, not mapping other databases linked to MIMIC-IV, and not covering some clinical modifiers and qualifiers by FHIR redefinitions [16]; SNOMED coverage restrictions [10]; manual FHIR mapping [39]; duplication of the mapping terms and the necessity to assess the need for a new FHIR profile versus continuing with the existing one [40]; LOINC codes for some observations (SNOMED could be used instead), no available code systems for many value sets, and lack of ubiquitous adoption of FHIR profiles due to the issues with SNOMED license [41]; requiring constant synchronization to the updates because the model was based on EBMonFHIR resources (have low maturity level and subject to changes) and impossibility of showing all guideline information in the FHIR resource format [42]; no textual structure due to lack of gold standard labels, not using syntax-based features for semantic representation, and elimination of some contextual information [35]; translation issues (from OMOP to preferred code systems of FHIR) [37].

#### Performance

Performance issues are related to obstacles in the efficient processing and retrieval of data that can compromise system performance, for example, the data are not processed within an acceptable response time [72].

According to the analyzed papers, performance issues include integration speed limitations due to transactional EHR [9], performance limitations [13], lack of sophisticated evaluation method [21], limited implementation assessment [30], performance validation issues and no validation for real questions [28], technical challenges [29], model’s limited functionality and lack of comprehensive specification [10], no evidence to attain a balance of functionality and portability [38], reduced response rate due to using a web-based questionnaire [39], no execution engine available for representation format [42], evaluation and validation [34], performance rate lower than others and low *F*_1_-score [35], evaluation issues (one instance of MIMIC-III OMOP CDM, no rigorous evaluation) and no comprehensive assessment [43].

#### Scalability

Scalability limitations can be considered as the data model’s weakness in handling increasing data volume or workload.

#### Generalizability

Generalizability problems are challenges in the applicability of the data model to other aspects or settings.

Considering the selected papers, scalability and generalizability limitations include few resources being used [15]; limited corpora reuse [16]; not enough compatibility and generalizability experiments [15]; not using a real environment [27]; possible bias when conducting a similar study and challenging generalizability for other types of a disease (in this case other cancers) [28]; restriction in the adaptability of the best-performing prediction model and requiring more complex methods to empower prediction and cover diversity [25]; the generalizability issues of the platform [32]; no broad adoption of the app due to issues related to resources and expertise, the best performance for specific programs [33]; few data models are used with no exhaustive evaluation [35]; low contribution to the medical field, failure to distinguish differences in genetic data, low resources for evaluation, and lack of comprehensive data modeling comparisons [36]; and not studying other test types and small sample size [43].

## Discussion

### Principal Findings

In this review, we aimed to provide a comprehensive PRISMA-based overview of data models using FHIR in the context of interoperability, structure, and functionality and summarize the state of the art for developing FHIR-based data models. In addition, we highlighted limitations, challenges, advantages, and opportunities brought about by FHIR data models. On the basis of the reviewed papers, the most common resources were from the “Clinical” (Observation and Condition) and “Base” (Patient resource) categories of FHIR resources. To develop the models, researchers focused more on the use cases, such as chronic diseases, cancer, COVID-19 and infectious diseases, and intensive care. The reason may be the availability of data in these fields. For instance, Mayo Clinic’s clinical data warehouses provide cancer data for researchers. Moreover, i2b2 and MIMIC offer health care data sets about chronic diseases such as obesity. MIMIC-IV on FHIR is also accessible for research in critical care, which provides deidentified FHIR-based data [26].

In terms of limitations, data integration issues are among the most significant challenges in developing data models. The necessity for manual ETL processes, the potential for information loss, and the use of constrained and incomplete data sets can impede the data integration process. Furthermore, organizational differences and hard-coded mappings complicate the seamless exchange of data, affecting interoperability. Issues, such as speed limitations and a lack of robust evaluation metrics, negatively impact performance. In addition, scalability and generalizability are further hindered by limited resources, insufficient compatibility experiments, and small sample sizes.

However, apart from the limitations and challenges, there are numerous advantages to using FHIR-based data models. This standard uses a set of resources and attributes (either common or unique) that enhance data modeling procedures. Constraining the attributes based on an adaptation of clinically relevant ontologies, such as *International Classification of Diseases, Ninth Revision (ICD-9),* and *International Classification of Diseases, Tenth Revision (ICD-10)*, LOINC, and SNOMED CT, is done through common data types (eg, codeable concepts and string). FHIR can be integrated with other data models, such as RDF, to provide a network-based model for disease prediction. It also supports feature generation and network population in these frameworks [25].

The use of the FHIR models provides the potential to significantly enhance the efficiency and effectiveness of health care research [9,15,29]. CRFs can be automatically populated with FHIR-based EHR data [21], and this automation can identify patient subgroups by topic modeling [28]. EHR data can also be harmonized and mapped to FHIR elements to enhance interoperability and quality of care [15]. Therefore, accessing health-related data for research would be more efficiently achieved when the data are in the FHIR format. Researchers and practitioners can access FHIR app galleries through FHIR APIs and SMART-on-FHIR applications, which can promote health care research and quality of care. FHIR also enables health care professionals to create use case–specific and customized applications [30]. However, implementing SMART-on-FHIR apps poses multiple dissemination challenges and barriers because FHIR-based APIs are not generally considered in the initial stages of implementation in some EHRs [38]. By contrast, as more EHRs choose FHIR as a data exchange standard, nonacademic health care settings will also tend to produce FHIR-formatted data with their EHR systems using FHIR APIs [23]. Maintaining health care data in the format of FHIR resources and using RESTful APIs provide more efficient data transmission compared to conventional record-keeping methods [31].

Another possibility is to use patient messages in web-based portals for health care research. In this respect, a FHIR data model can be developed to extract essential information and concepts from this type of data; FHIR is a beneficial option because it encapsulates modular actions and concepts in health information sharing [34]. Moreover, FHIR exports of local data repositories increase data interoperability for systems and data warehouses [22]. Using FHIR ensures standardized data representation, supports data quality through validation tools (eg, IGs and FHIR specifications), offers flexible adaptation, and benefits from strong community support [35]. The transition of traditional medical guidelines to machine-readable FHIR IGs is a sophisticated and advancing process and needs validation approaches. These use case–specific IGs can eventually enhance real-world application and interoperability of the clinical guidelines [40], considering the constant feedback and inputs from related health care communities [43]. Furthermore, it is crucial to thoroughly follow domain-specific FHIR IGs to gain optimal semantic interoperability. However, even with this guidance provided by IGs, developers still have to make numerous representation and implementation decisions, which may not always be ideal [33].

Evidence-based and computer-interpretable guidelines can be developed using structured data from frameworks’ evidence and reviews, followed by mapping the derived items to EBM-on-FHIR resources. This approach aligns with the CPG-on-FHIR framework and includes FHIR profiles and IGs [42].

On the basis of the results of a research paper on using the FHIR standard in oncology, specific health care domains can also be modeled with minimal gaps between FHIR and other standards using annotations of embedded mappings [41]. In addition, in the field of EHR phenotyping and data capture, using FHIR-based data normalization pipelines is considered valuable and beneficial [13]. Using FHIR-based NLP extensions and FHIR composition resources represents NLP components in phenotyping algorithms [20]. Inherently, as mentioned earlier, FHIR resources have granular and atomic characteristics that enable them to share only the required elements for specific use cases and purposes rather than a wide range of elements. This feature is useful for developing specialized AI platforms and interpreting machine learning algorithms [10]. By contrast, this multilayer and complex structure of FHIR may cause some accessibility issues for AI algorithms. To be useful for AI, FHIR data often need to be transformed into a simpler format with a higher level of abstraction, making it more compatible with typical data preprocessing tools. This transformation seems to be necessary to make the data more manageable and easier to analyze by AI systems [15].

Using machine learning algorithms and NLP models is advantageous when integrating with FHIR data models and structures [12] in cases such as standardizing heterogeneous corpora [16]. FHIR modeling for EHR data enhances data integration, data transmission, translational research, and phenotyping [12]. Similarly, the NLP2FHIR pipeline can be used to enhance the standardization of unstructured EHR data [14]. The researchers illustrated how deep learning models can be effectively transferred and used across different settings or systems when dealing with data that have been processed using NLP2FHIR representations. This pipeline performed better in text classification in comparison with models using original texts [35].

### Limitations

This review has some limitations. First, we focused only on the articles that dealt with specific use cases with real-world data. By contrast, this approach enabled us to gain insight into the practical applications of the subject matter in real-world contexts rather than merely theoretical ones. In this review, we may have some generalizability issues or biases, especially in the resources and methodologies used and with ontological, general, and theoretical data models. Second, we did not analyze articles published in languages other than English, so we potentially missed some articles due to this criterion. Third, because we did not thoroughly analyze the gray literature and preprints, and due to the relatively small number of included articles, some results may not be generalizable to the entire field of FHIR data modeling.

### Comparison With Prior Work

There are some valuable review studies considering the FHIR standard (Table 4). In a scoping review, Balch et al [58] investigated machine learning–based clinical information systems that used the FHIR standard. The focus of the review was to analyze analytics and data management platforms, CDSSs, and APIs and assess the systems’ functionalities as well as strengths and weaknesses. Then, the researchers proposed a clinical structure that integrated FHIR and machine learning techniques.

**Table 4:** The summary of previous reviews considering FHIR^a^.

Study, year	Title	Items included in the review
Balch et al [58], 2023	Machine Learning–Enabled Clinical Information Systems Using Fast Healthcare Interoperability Resources Data Standards: Scoping Review	Investigation of FHIR-based systems using machine learning methods focusing on decision support, data analytics, and APIs^b^ PRISMA-ScR^c^ guideline’s steps Categorization of the articles based on functionalities, limitations, and strengths Proposing a machine learning–based system using FHIR
Nan and Xu [59], 2023	Designing Interoperable Health Care Services Based on Fast Healthcare Interoperability Resources: Literature Review	Reviewing FHIR-based studies about interoperable health services Study year and country distributions and charts Flowchart of paper selection Clinical categorization of studies and corresponding FHIR resources Data model migrations to FHIR Data management methods Data integration modes Presenting a FHIR practice design and its development architecture Commonly used tools
Pimenta et al [60], 2023	Interoperability of Clinical Data through FHIR: A review	Some selected examples and applications of FHIR (data standards, analysis, API implementations, and research) PRISMA^d^ chart
Pavão et al [61], 2023	The Fast Health Interoperability Resources (FHIR) Standard and Homecare, a Scoping Review	Home care research studies focusing on FHIR Screening and inclusion details FHIR resources Technological tools in the implementation phase Privacy and security measures
Ayaz et al [62], 2021	The Fast Health Interoperability Resources (FHIR) Standard: Systematic Literature Review of Implementations, Applications, Challenges and Opportunities	Focusing on FHIR and EHR^e^; all articles dealt with FHIR related to research questions Screening and inclusion details Study year, type, and country distributions Primary subject categories over time FHIR resource list Types of applications that leverage FHIR Data mappings form or to FHIR Objectives of using FHIR Challenges of using FHIR
Vorisek et al [63], 2022	Fast Healthcare Interoperability Resources (FHIR) for Interoperability in Health Research: Systematic Review	FHIR-based implementations in health care research PRISMA flowchart for article inclusion Study year distribution and coauthorship network chart Research domains FHIR applications, international standards, and medical domain FHIR resources Items mapped to FHIR Objectives of using FHIR Limitations of using FHIR
Duda et al [64], 2022	HL7^f^ FHIR-based tools and initiatives to support clinical research: a scoping review	Trends and gaps in using FHIR in health care research PRISMA flowchart for article and project inclusion The expansion of Marquis-Gravel categorization [43] of FHIR-based projects contributing to research, in the categories of preparation, prestudy, study setup, recruitment, study conduct, and poststudy activities Gaps in using FHIR in clinical research
Lehne et al [65], 2019	The Use of FHIR in Digital Health - A Review of the Scientific Literature	Investigation of using FHIR in digital health care Article selection flowchart Study year and article category distribution charts Abstract text mining for most frequent words
Yogesh and Karthikeyan [66], 2022	Health Informatics: Engaging Modern Healthcare Units: A Brief Overview	FHIR architecture in health care units Narrative explanation of FHIR definitions, FHIR data layers and resources, and workflow relations Health informatics challenges, some related to FHIR
Schweitzer et al [67], 2022	Data Exchange Standards in Teleophthalmology: Current and Future Developments	Interoperability standards in the field of store-and-forward ophthalmology Reviewing IHE^g^, HL7 standards, DICOM^h^, and health care terminologies
Torab‑Miandoab et al [68], 2023	Interoperability of heterogeneous health information systems: a systematic literature review	Interoperability in heterogeneous health care systems PRISMA flowchart for article selection Charts and figures for frequencies and trends of interoperability articles Summary and categorization of interoperability standards and architecture components Word cloud figures for frequent standards and platforms Interoperability levels

^a^FHIR: Fast Healthcare Interoperability Resources.

^b^API: application programming interface.

^c^PRISMA-ScR: Preferred Reporting Items for Systematic Reviews and Meta-Analyses extension for Scoping Reviews.

^d^PRISMA: Preferred Reporting Items for Systematic Reviews and Meta-Analyses.

^e^EHR: electronic health record.

^f^HL7: Health Level 7.

^g^IHE: Integrating the Healthcare Enterprise.

^h^DICOM: Digital Imaging and Communications in Medicine.

Nan and Xu [59] reviewed FHIR-related papers on designing and building interoperable health care services with a focus on data standardization, management, and integration. The researchers analyzed detailed processes and techniques for each group, resulting in a comprehensive FHIR practice guideline. Similar to our research, Nan and Xu [59] reviewed important techniques and FHIR resources for developing health care services. The difference between this research and our study is the focus of the research; they [59] investigated a broader range of FHIR-based studies and services, while we focused more on data models and infrastructures.

Pavão et al [61] reviewed research articles using the FHIR standard in home care services. The researchers aimed to analyze FHIR resources, types of home care applications, privacy and security considerations, and deployment tools. Pimenta et al [60] reviewed interoperability with FHIR and summarized some important points and examples. The researchers selected some articles and extracted FHIR applications in each study. Ayaz et al [62] in 2021 reviewed all aspects of FHIR in the articles published from January 2012 to December 2019. Our study also considered more recent articles from 2020 to 2023. The main objective of their study was to analyze the articles according to the implementation, challenges, future applications, and opportunities of this standard. The researchers reviewed articles that focused on all categories, including apps, FHIR implementation models, FHIR resources, FHIR framework, mapping framework and data model, challenges, and FHIR goals. The researchers also summarized the resources used in the included articles; “Observation” and “Patient” resources, respectively, were the most commonly used resources in the included articles. We also performed this analysis and had close results; as we mentioned earlier in our study, “Observation,” “Condition,” and “Patient” resources were used more frequently. The mentioned researchers also discussed the mapping approaches from other techniques or methods to FHIR. The focus of the study by Vorisek et al [63] was to review the FHIR standard from a “health research” perspective. The researchers analyzed the studies that used FHIR in any aspect of the research process, such as data collection, recruitment, data standardization, analysis, and consent management. In addition, they categorized the articles with generic or specific clinical specialty approaches. We also categorized our articles based on the medical field. In the mentioned research, it was reported that most studies used other terminologies and data models besides FHIR, which included SNOMED CT, LOINC, International Classification of Diseases 10th Revision, OMOP CDM, and more. It was reported that among “data capture–related” studies, the “Questionnaire” resource was used more frequently, as expected. In addition to the scientific aspects, the limitations of using FHIR were similarly discussed. They highlighted that the limitations may include the evolving contents of FHIR resources, legal issues, safety, and the need to have an FHIR server. In our study, by contrast, we categorized the limitations into other aspects.

Regarding the medical research aspects of FHIR, Duda et al [64] also presented a literature review. The study extended the “Marquis-Gravel categories” [73], in which it is possible to categorize the way each project contributes to research tasks. The FHIR projects focused on research were investigated, which included the activities of preparation (eg, mapping to and from FHIR), prestudy (eg, defining or refining of cohorts), study design (eg, data collection for research), recruitment (eg, including screening criteria in EHR), study conduct (eg, patient data collection), and poststudy (eg, data sharing). Most projects focused on “general research preparation” (eg, infrastructure and data pipeline development). Lehne et al [65] reviewed the application of FHIR in digital health. On the basis of their research, the reviewed articles were mostly related to data models, mobile or web applications, and medical devices. Yogesh and Karthikeyan [66] reviewed the FHIR architectural specifications, such as the linkages, workflow state, health informatics, and public health safety approaches using this standard. The researchers also highlighted the likely challenges with health care data standards, including coding speed and accuracy issues, code mappings, compatibility issues between new and legacy systems, and communication concerns between EHRs and patients.

Some other articles reviewed general interoperability and data exchange standards. In the research conducted by Schweitzer et al [67], the researchers narratively described and compared exchange approaches, such as Digital Imaging and Communications in Medicine (DICOM), the Integrating the Health Care Enterprise initiative, and clinical terminologies (such as SNOMED CT) as well as FHIR in the field of teleophthalmology. In their research, the ophthalmology-related FHIR resource, which is “VisionPrescription,” as well as the current proposal of the related IG were discussed. Torab‑Miandoab et al [68] reviewed interoperability approaches and requirements for semantic interoperability between heterogeneous health information systems. It was found that FHIR, Clinical Document Architecture (CDA), Service-Oriented Architecture, Reference Information Model, Health Insurance Portability and Accountability Act security act, SNOMED CT, XML, JAVA, SQL, and API can be considered the most important requirements to implement semantic interoperability. On the basis of the results, a summary of interoperability standards in the context of terminology, content, transport, and security was also presented. The researchers highlighted the categorization of interoperability architecture components with the main categories of service-oriented architecture, archetype-based, web-based, client-server, multiagent, blockchain-based, XML-based, cloud-based, ontology-based, object-oriented, and local network.

### Future Directions and Recommendations

In the course of this study, we encountered some ideas and recommendations for future research. These included the following: (1). a comparison of health care data models with the use of FHIR and other standards, including earlier versions of HL7 interoperability standards (such as HL7-version 2 and version 3 and CDA), OpenEHR, and OMOP CDM. The aim would be to provide a detailed analysis of the models created with these standards, focusing on the methodological aspects, limitations, strengths, and maintenance of interoperability. (2). an examination of the ontological aspects of data models and a discussion of how they represent medical terminologies and concepts.

### Conclusions

FHIR serves as a highly promising interoperability standard for developing real-world health care applications. The integration of FHIR with other data models facilitates the development of more interoperable domain-specific solutions and improves research efficiency. In addition, the implementation of FHIR modeling for EHR data facilitates the integration, transmission, and analysis of data while also advancing translational research and phenotyping. Several FHIR data models have been developed to enhance the extraction of essential information and concepts from unstructured data such as patient summaries retrieved from EHRs. Generally, FHIR-based exports of local data repositories improve data interoperability for systems and data warehouses across different settings. However, ongoing efforts to address existing limitations and challenges are essential for the successful implementation and integration of FHIR data models.

## Appendix

Multimedia Appendix 1 PRISMA-ScR (Preferred Reporting Items for Systematic Reviews and Meta-Analyses extension for Scoping Reviews) checklist.

Multimedia Appendix 2 PRISMA-P (Preferred Reporting Items for Systematic Review and Meta-Analyses Protocols) 2015 checklist and review protocol.

Multimedia Appendix 3 Search strategy.

## Abbreviations

AI: artificial intelligence
API: application programming interface
CASIDE: Cancer Survivorship Interoperable Data Elements
CPG: clinical practice guideline
CQL: Clinical Quality Language
CRF: case report form
cTAKES: Clinical Text Analysis and Knowledge Extraction System
DICOM: Digital Imaging and Communications in Medicine
DKTK: German Cancer Consortium (From German)
EBMonFHIR: Evidence-Based Medicine–on-FHIR
EHR: electronic health record
ETL: Extract, Transform, and Load
FASTO: Fast Healthcare Interoperability Resources and Semantic Sensor Network-Based Type 1 Diabetes Ontology
FHIR: Fast Healthcare Interoperability Resources
GCN: graph convolutional network
HAPI: Health Level 7 application programming interface
HIMSS: Healthcare Information and Management Systems Society
HL7: Health Level 7
IG: implementation guide
KG: knowledge graph
LOINC: Logical Observation Identifiers Names and Codes
MedXN: Medication Extraction and Normalization
MIMIC: Medical Information Mart for Intensive Care
NLP: natural language processing
OMOP CDM: Observational Medical Outcomes Partnership−Common Data Model
PRISMA: Preferred Reporting Items for Systematic Reviews and Meta-Analyses
PRISMA-P: Preferred Reporting Items for Systematic review and Meta-Analysis Protocols
PRISMA-ScR: Preferred Reporting Items for Systematic Reviews and Meta-Analyses extension for Scoping Reviews
QUICK: Quality Improvement and Clinical Knowledge
RDF: Resource Description Framework
SMART: Substitutable Medical Apps, Reusable Technology
SNOMED CT: Systemized Nomenclature of Medicine−Clinical Terms
SVM: support vector machine
