# Supplementary material for: State-of-the-Art Fast Healthcare Interoperability Resources (FHIR)–Based Data Model and Structure Implementations: Systematic Scoping Review
Source: JMIR Med Inform. 2024 Sep 24;12:e58445. doi: 10.2196/58445 (PMC11472501; doi:10.2196/58445)
Supplement: Multimedia Appendix 2 [file medinform_v12i1e58445_app2.pdf]

PRISMA-P (Preferred Reporting Items for Systematic review and Meta-Analysis Protocols) 2015 checklist: recommended items to address in a systematic review protocol*

Section and topic	Item No	Checklist item
**ADMINISTRATIVE INFORMATION**		
Title:		
Identification	1a	Identify the report as a protocol of a systematic review
Update	1b	If the protocol is for an update of a previous systematic review, identify as such
Registration	2	If registered, provide the name of the registry (such as PROSPERO) and registration number
Authors:		
Contact	3a	Provide name, institutional affiliation, e-mail address of all protocol authors; provide physical mailing address of corresponding author
Contributions	3b	Describe contributions of protocol authors and identify the guarantor of the review
Amendments	4	If the protocol represents an amendment of a previously completed or published protocol, identify as such and list changes; otherwise, state plan for documenting important protocol amendments
Support:		
Sources	5a	Indicate sources of financial or other support for the review
Sponsor	5b	Provide name for the review funder and/or sponsor
Role of sponsor or funder	5c	Describe roles of funder(s), sponsor(s), and/or institution(s), if any, in developing the protocol
**INTRODUCTION**		
Rationale	6	Describe the rationale for the review in the context of what is already known
Objectives	7	Provide an explicit statement of the question(s) the review will address with reference to participants, interventions, comparators, and outcomes (PICO)
**METHODS**		
Eligibility criteria	8	Specify the study characteristics (such as PICO, study design, setting, time frame) and report characteristics (such as years considered, language, publication status) to be used as criteria for eligibility for the review
Information sources	9	Describe all intended information sources (such as electronic databases, contact with study authors, trial registers or other grey literature sources) with planned dates of coverage
Search strategy	10	Present draft of search strategy to be used for at least one electronic database, including planned limits, such that it could be repeated
Study records:		

Data management	11a	Describe the mechanism(s) that will be used to manage records and data throughout the review
Selection process	11b	State the process that will be used for selecting studies (such as two independent reviewers) through each phase of the review (that is, screening, eligibility and inclusion in meta-analysis)
Data collection process	11c	Describe planned method of extracting data from reports (such as piloting forms, done independently, in duplicate), any processes for obtaining and confirming data from investigators
Data items	12	List and define all variables for which data will be sought (such as PICO items, funding sources), any pre-planned data assumptions and simplifications
Outcomes and prioritization	13	List and define all outcomes for which data will be sought, including prioritization of main and additional outcomes, with rationale
Risk of bias in individual studies	14	Describe anticipated methods for assessing risk of bias of individual studies, including whether this will be done at the outcome or study level, or both; state how this information will be used in data synthesis
Data synthesis	15a	Describe criteria under which study data will be quantitatively synthesised
	15b	If data are appropriate for quantitative synthesis, describe planned summary measures, methods of handling data and methods of combining data from studies, including any planned exploration of consistency (such as I^2, Kendall's τ)
	15c	Describe any proposed additional analyses (such as sensitivity or subgroup analyses, meta-regression)
	15d	If quantitative synthesis is not appropriate, describe the type of summary planned
Meta-bias(es)	16	Specify any planned assessment of meta-bias(es) (such as publication bias across studies, selective reporting within studies)
Confidence in cumulative evidence	17	Describe how the strength of the body of evidence will be assessed (such as GRADE)

*** It is strongly recommended that this checklist be read in conjunction with the PRISMA-P Explanation and Elaboration (cite when available) for important clarification on the items. Amendments to a review protocol should be tracked and dated.** **The copyright for PRISMA-P (including checklist) is held by the PRISMA-P Group and is distributed under a Creative Commons Attribution Licence 4.0.**

From: Shamseer L, Moher D, Clarke M, Ghersi D, Liberati A, Petticrew M, Shekelle P, Stewart L, PRISMA-P Group. Preferred reporting items for systematic review and meta-analysis protocols (PRISMA-P) 2015: elaboration and explanation. BMJ. 2015 Jan 2;349(jan02 1):g7647.

Administrative Information:

Title:
Identification: State-of-the-Art FHIR–Based Data Model and Structure Implementations: Systematic Scoping Review Protocol

Authors:
Parinaz Tabari[1*], Gennaro Costagliola[1], Mattia De Rosa[1], Martin Boeker[2]

[1] Department of Informatics, University of Salerno, Fisciano, Italy
[2] Institute for Artificial Intelligence and Informatics in Medicine, Medical Center rechts der Isar, School of Medicine and Health, Technical University of Munich, Munich, Germany
[*] Drafted the protocol

Support: Partial funding by the German Federal Ministry of Education and Research within the Medical Informatics Initiative DIFUTURE FKZ 01ZZ2304A.

Introduction:

Rationale: To the best of our knowledge, no research has been done so far to comprehensively assess the practical implementations of FHIR-based models and infrastructures. Thus, in this research, we aim to review recent advancements in this field, focusing on the functional data models or infrastructure/framework implementations using this standard. More specifically, this scoping review focuses on addressing the question "What insights can be gained from analyzing the state-of-the-art FHIR-based data modeling approaches considering technological advancements, application in the medical domain, and potential limitations?"

Objectives: The research objectives are as follows: (1) to provide a comprehensive overview of FHIR-based data models in the context of interoperability, structure, and functionality and summarize the state of the art for developing FHIR-based data models and (2) to highlight limitations, challenges, advantages, and opportunities brought about by FHIR-based data models

Methods:

Conducting the review according to the instructions of the Preferred Reporting Items for Systematic reviews and Meta-Analyses extension for Scoping Reviews (PRISMA-ScR) checklist.
This checklist aims to facilitate the development of a deeper comprehension of pertinent terminology, fundamental concepts, and essential items to report for scoping reviews.

Eligibility Criteria:

To select the papers, we consider the articles that encompass the FHIR standard in the data model development or infrastructure design. The inclusion and exclusion criteria are defined as follows in more detail:

Inclusion Criteria:

- Original articles and case studies (journals - conferences)
- Articles related to the FHIR-based data models and structures focusing on a health condition or using real-world patient data, registries, or data sets
- Articles with high-quality and detailed workflow processes with at least one architecture/data model diagram
- Articles that discuss the barriers, challenges, or limitations of developing FHIR-based data models and infrastructures in a health domain

Exclusion Criteria:

- Not written in English
- Not accessible full text
- Letter to the editors, reviews, editorials, commentary articles, short papers without detailed implementation information, posters, and preprint articles
- Not relevant to research questions and objectives; in other words, articles not focusing on FHIR-based data model development or not providing practical and detailed insights into the development or use of FHIR-based data models by a schematic approach
- Papers lacking specific use cases or real-world data sources or without discussion of limitations and challenges

Information Sources: Academic databases, such as PubMed, Scopus, Web of Science (standard selection of databases—Web of Science Core Collection), IEEE Xplore, and the ACM Digital Library.
It is worth mentioning that no time limit will be applied to the search to obtain a comprehensive overview of all published articles in this field. We should clarify that the initial pages of Google Scholar (9-10 pages) will be investigated as a supplement to the mentioned academic libraries to retrieve additional papers.

Search Strategy:
Database-specific variants of the basic search term ((”fhir”) AND (”data model” OR ”modelling” OR ”minimum data set” OR “data element”)) with their synonyms, variations, and full forms.
Scopus Search: (TITLE-ABS-KEY(“fhir” OR “fast healthcare interoperability resources”) AND (TITLE-ABS-KEY(“data model” OR “modelling” OR “modeling” OR “MDS” OR “minimum data set” OR “minimum dataset” OR “data element*”)))

Study Records:

In a stepwise process, 2 co-authors (PT and MDR) will independently screen the retrieved articles and select the initial studies by applying the inclusion and exclusion criteria to the titles or abstracts or, in some cases, full texts (by rapid skimming).
Inconsistencies in the selection will be discussed with other co-authors until a consensus is reached.
The full texts of the initially selected articles will be assessed in the next phase to check compliance with the eligibility criteria. PT will thoroughly review the articles and then discuss them with other authors about inclusions.
Disagreements will be resolved after group discussions. Each selected study will be thoroughly investigated for the appropriateness and clarity of the research methodology and design. We will also assess them to ensure alignment with the study objectives. The rigor of the methods, tools, and techniques employed for FHIR-based architectural design will be considered in this phase. The presentation of results and the coherence of model interpretation will also be closely examined.

Data management: EndNote X9 software for article screening and investigation in each step will be used.

Data collection process: This will be done by thoroughly reviewing the articles and then discussing them with other authors about inclusions. Disagreements will be resolved after group discussions.

Data items:

Bibliographic information, such as title, authors, and year of publication
Data sources
FHIR resources
Data transformation and mapping
Standards/tools/terminologies/models
Data validation/evaluation
Use case

Outcomes and prioritization: The rigor of the methods, tools, and techniques employed for FHIR-based architectural design, and compliance with inclusion and exclusion criteria will be assessed. The presentation of results and the coherence of model interpretation will be examined.

Data Synthesis:
After extracting the information, we will assess them to find themes or categories. Subsequently, we will perform a general analysis of the papers, based on the overall technical themes, and the medical domains. Additionally, any important technologies used most in the included articles will be comprehensively presented and discussed afterward. Resource frequency analysis will be performed via the investigation and counting of FHIR resources utilized in each data model and infrastructure to find out which resources are more common in system developments. One of the most important aims of our research is to extract and categorize the implementation limitations mentioned by the researchers. Therefore, these aspects will also be addressed subsequently to provide a thorough viewpoint of challenges that future scientists may face.
