# Supplementary material for: State-of-the-Art Fast Healthcare Interoperability Resources (FHIR)–Based Data Model and Structure Implementations: Systematic Scoping Review
Source: JMIR Med Inform. 2024 Sep 24;12:e58445. doi: 10.2196/58445 (PMC11472501; doi:10.2196/58445)
Supplement: Multimedia Appendix 3 [file medinform_v12i1e58445_app3.pdf]

Database	Search strategy	Retrieved articles (N.)
Scopus	(TITLE-ABS-KEY("fhir" OR "fast healthcare interoperability resources")) AND (TITLE-ABS-KEY("data model" OR "modelling" OR "modeling" OR "MDS" OR "minimum data set" OR "minimum dataset" OR "data element*"))	175
PubMed	(((((((data model[Title/Abstract]) OR (modelling[Title/Abstract])) OR (modeling[Title/Abstract])) OR (MDS[Title/Abstract])) OR (minimum data set[Title/Abstract])) OR (minimum dataset[Title/Abstract])) OR (data element*[Title/Abstract])) AND ((fhir[Title/Abstract]) OR (fast healthcare interoperability resources[Title/Abstract]))	114
WoS	AB=("fhir" OR "fast healthcare interoperability resources") AND AB=("data model" OR "modelling" OR "modeling" OR "MDS" OR "minimum data set" OR "minimum dataset" OR "data element*")	59
IEEE Xplore	(("fhir" OR "fast healthcare interoperability resources") AND ("data model" OR "modelling" OR "modeling" OR "MDS" OR "minimum data set" OR "minimum dataset" OR "data element*"))	17
ACM	[[Abstract: "fhir"] OR [Abstract: "fast healthcare interoperability resources"]] AND [[Abstract: "data model"] OR [Abstract: "modelling"] OR [Abstract: "modeling"] OR [Abstract: "mds"] OR [Abstract: "minimum data set"] OR [Abstract: "minimum dataset"] OR [Abstract: "data element*"]]	10
Google Scholar	*(("fhir" OR "fast healthcare interoperability resources") AND ("data model" OR "modelling" OR "modeling" OR "MDS" OR "minimum data set" OR "minimum dataset" OR "data element*"))*	*91*
